# The effect of cognitive dysfunction on mid- and long-term mortality after vascular surgery

**DOI:** 10.1186/s12877-020-01994-x

**Published:** 2021-01-13

**Authors:** András Szabó, Krisztina Tóth, Ádám Nagy, Dominika Domokos, Nikoletta Czobor, Csaba Eke, Ágnes Sándor, Béla Merkely, Éva Susánszky, János Gál, Andrea Székely

**Affiliations:** 1 Department of Anesthesiology and Intensive Therapy, Semmelweis University, 78 Üllői st., Budapest, H-1082 Hungary; 2 Károly Rácz School of PhD Studies, Semmelweis University, Budapest, Hungary; 3 Heart and Vascular Center, Semmelweis University, Budapest, Hungary; 4 Department of Anesthesiology and Intensive Care, Medical Centre of Hungarian Defense Forces, Budapest, Hungary; 5 Institute of Behavioural Science, Semmelweis University, Budapest, Hungary

**Keywords:** Vascular surgery, Perioperative risk factors, Cognitive dysfunction, Mini mental state examination, Social support, Psychosocial factors

## Abstract

**Background:**

In recent decades, previous studies have noted the importance of frailty, which is a frequently used term in perioperative risk evaluations. Psychological and socioeconomical domains were investigated as part of frailty syndrome. The aim of this study was to assess the importance of these factors in mortality after vascular surgery.

**Methods:**

In our prospective, observational study (ClinicalTrials.gov Identifier: NCT02224222), we examined 164 patients who underwent elective vascular surgery between 2014 and 2017. At the outpatient anaesthesiology clinic, patients completed a questionnaire about cognitive functions, depression and anxiety, social support and self-reported quality of life were assessed using a comprehensive frailty index, in addition to medical variables. Propensity score matching was performed to analyse the difference between patients and controls in a nationwide population cohort. The primary outcome was 4 year mortality. The Kaplan-Meier method and Cox regression analysis were used for statistical analyses.

**Results:**

The patients’ mean age was 67.05 years (SD: 9.49 years). Mini-Mental State Examination scores of less than 27 points were recorded for 41 patients. Overall mortality rates were 22.4 and 47.6% in the control and cognitive impairment groups, respectively (*p* = 0.013). In the univariate Cox regression analysis, cognitive impairment measured using age- and education-adjusted MMSE scores increased the risk of mortality (AHR: 2.842, 95% CI: 1.389-5.815, *p* = 0.004).

**Conclusion:**

Even mild cognitive dysfunction measured preoperatively using the MMSE represents a potentially important risk factor for mortality after vascular surgery.

## Background

In the preoperative period, the ideal situation would be to identify all potential risk factors that might cause adverse events and/or negatively affect the outcome. Frailty syndrome is an age-related, multidimensional state of decreased physiological reserve that results in diminished resiliency and increased vulnerability of patients. Frailty has been proven to be an excellent covariate of unfavourable health outcomes in the older surgical population [1]. It has been studied intensively for the past two decades, and frail patients undergoing surgery appear to have significantly worse short- and mid-term survival rates than the nonfrail patient population [2, 3].

According to the modern and comprehensive definition, frailty is a medical syndrome with multiple causes and contributors that is characterized by diminished strength and endurance and reduced physiological function, leading to increased vulnerability to adverse health outcomes such as functional decline and early mortality [4]. This general concept encompasses all the factors with serious effects on mortality and quality-adjusted life years.

Based on clinical experience, some other factors that are not routinely evaluated might influence postoperative outcomes. Frailty has refined the former risk stratification based on clinically measured and previous medical data, enabling a more precise assessment of the length and difficulty of healing and recovery after surgery. Traditionally, an older age, lower educational level, current smoking status, current use of postmenopausal hormone therapy, certain ethnicities, an unmarried state, clinical depression or use of antidepressants, and intellectual disability are mentioned as the most important cofactors of frailty [5–8]. From this list, frailty risk factors are basically divided into two main categories. In this current article, we focus on factors including cognitive, mental, social and psychological aspects rather than the traditional scoring system.

### Objectives

This study aimed to preoperatively identify the most important psychological and social variables that may influence postoperative outcomes in patients undergoing vascular surgery.

The primary endpoint was overall mortality. A comparison between our patient population undergoing vascular surgery and a representative control population cohort was also performed to identify the potential differences in psychosocial attitudes.

## Methods

### Study design, setting, participants

This study was approved by the Regional Ethics Committee (TuKEB 250/2013) and registered at ClinicalTrials.gov (NCT02224222). The inclusion criteria were as follows: age over 18 years, native Hungarian speaker and undergoing elective vascular surgery. Exclusion criteria were pregnant women and patients with a legal incapacity or considered to have a limited capability to understand the study procedures and provide ethical consent. All clients were capable of making decisions regarding their participation in this study, and accordingly, written consent was obtained. A study nurse, a medical student or a postdoctoral fellow invited patients to participate in the study during their outpatient anaesthesiology visit. Every person of the enrolled staff was trained by a psychologist to perform correct cognitive mapping and assessments. Baseline questionnaires were completed 5–30 days before surgery. After signing the informed consent form, 199 adult patients were enrolled prospectively at the Department of Vascular Surgery of the Heart and Vascular Center of Semmelweis University in Budapest between September 2014 and August 2017. Thirty-two patients were excluded because of cancelled surgery. Three patients withdrew their consent. Finally, data from 164 patients were used for the statistical analysis.

### Definitions and measurements (variables, data sources, and grouping)

Wide ranges of clinical and psychosocial factors were assessed as potential determinants of the outcome. Clinical factors included perioperative laboratory parameters (blood counts, renal function measures, ion levels, etc.), intraoperative parameters (operation time, cross-clamp time, blood loss, need for transfusions and fluid balance medications), postoperative parameters (blood loss, medications, etc.), outcomes, the incidence and severity of postoperative complications (major cerebrovascular or neurological event; acute or chronic heart failure defined as pulmonary oedema, atrial fibrillation, arrhythmias, cyanosis, metabolic disorders, need for inotropes, respiratory failure; infection; acute renal failure/need for renal replacement therapy; length of mechanical ventilation; length of ICU and in-hospital stay and in-hospital mortality rate). The American Society of Anesthesiologists risk score (ASA score) [9] and the Vascular Physiological and Operative Severity Score for the enUmeration of Mortality and Morbidity (vascular POSSUM) [10–13] were also calculated. The vascular-POSSUM consists of two parts, a physiological score and an operative score. The physiological score includes age and major vital parameters (cardiac, renal, haematological and neurological function), and the operative score focuses on intraoperative blood loss, peritoneal contamination, possible malignancy and the length and urgency of the procedure.

### Psychosocial factors

Psychosocial and demographic data were collected, e.g., age, sex, living conditions, smoking, alcohol consumption and education. Then, participants were asked to complete many questionnaires measuring psychosocial factors: the Beck Depression Inventory (BDI), the Spielberger State and Trait Anxiety Inventory (STAI-S and STAI-T), the Mini Mental State Examination (MMSE), the Geriatric Depression Scale, the Somatic Symptom Severity Scale, the Devins Illness Intrusiveness Rating Scale, the Caldwell Social Support Dimension Scale, and specific parts of the Hungarostudy Query (a representative national study conducted in 2013 that was used as a control group and measured the health status, illnesses, biopsychosocial background and health-related quality of life (HRQ)).

For mapping, the Mini-Mental State Examination (MMSE) was applied to assess cognitive function. The MMSE is a well-established scale to screen cognitive deficits and signs of dementia. It contains simple questions and problems in many areas, including temporal-spatial orientation, short-term memory, arithmetic computation such as decreasing serial sevens, language use and comprehension, as well as basic visual-motor skills. The questionnaire score ranged from 0 to 30 points. Cut-off values are 23, 18 and 9 points for mild, moderate and severe cognitive impairment, respectively [14, 15]. In addition to an assessment of the raw MMSE results, adjustment for age and education level was performed. Patients with cognitive impairment were defined when a difference greater than 2 standard deviations between expected (age and education level adjusted) and MMSE scores was observed [14]. Modified cut-off values were used to detect the mildest cognitive impairment, according to previous publications [16–18]. In these studies, a cut-off value of 27 or lower indicated mild cognitive impairment, and a score of 23 or lower indicated a severe cognitive impairment.

Patients were asked to estimate self-reported happiness and satisfaction using a 1 to 10 point scale. These self-reported parameters were reported as an important aspect determining the long-term mortality of healthy adult volunteers [19].

The State-Trait Anxiety Inventory (STAI) was used to characterize the anxiety of patients. The inventory consists of two parts, the STAI-S and the STAI-T. The first 20 questions refer to the transitional emotional status evoked by a stressful situation (STAI-S), e.g., a hospital admission or a surgical intervention. The STAI-T score reflects personal differences in chronic anxiety susceptibility. Each group is scored from 20 to 80 points based on four-level Likert items [20, 21]. The STAI, a test with high reliability and validity, is well documented in the Hungarian population [22]. (STAI-T and S Cronbach’s α = 0.638 and 0.763, respectively).

The Beck Depression Inventory (BDI) was used for affective disorders. The BDI, a 21-item questionnaire, is an established tool for screening depression, with each item evaluating different symptoms of depression, such as a bad mood, pessimistic outlook, feelings of guilt and loss of appetite. Each item contains four sentences indicating the degree of severity for that particular symptom. Answers are four-level Likert items; the whole inventory is scored from 0 to 60 points [23–25]. The validity and reliability of the BDI are also well documented in the Hungarian population (Cronbach’s α = 0.787) [26].

The Geriatric Depression Scale is a yes-or-no question-based, 30-item inventory for the assessment of depression occurring in the older population. In our study, the short form of the GDS was used, which includes 15 questions. Every question is scored either 0 or 1, and the sum normally ranges from 0 to 9 points (Cronbach’s α = 0.704) [27].

The Somatic Symptom Severity Scale (Patient Health Questionnaire – PHQ15) refers to different symptoms, e.g., gastrointestinal dysfunction, dizziness, chest pain and dyspnoea. It is calculated by assigning scores of 0, 1 and 2 to the response categories of “not at all”, “bothered a little”, and “bothered a lot”, respectively, for all 13 somatic symptoms. Additionally, 2 items from the mood module (fatigue and sleep) are scored 0 (“not at all”), 1 (“several days”) or 2 (“more than half the days” or “nearly every day”). We did not use questions regarding pain caused by menstruation or dysmenorrhea for better comparability. Thus, the inventory is scored from 0 to 28 points. Scores of 5, 10, and 15 represent cut-off points for low, medium, and high somatic symptom severity, respectively (Cronbach’s α = 0.730) [28–30].

The Devins Illness Intrusiveness Rating Scale measures the effect of illness on different social issues. The 13-item questionnaire was introduced to screen for illness-induced disruptions in lifestyle, activities and interests that may compromise psychosocial well-being and contribute to emotional distress in patients with chronic diseases. Answers are seven-level Likert items; the inventory is scored from 13 to 91 points (Cronbach’s α = 0.854) [31, 32].

For the analysis of the patient’s social web structure, the Caldwell Social Support Dimension Scale was used. This scale is a novel version of the Social Support Questionnaire published originally in 1987 [33]. The intensity of different interpersonal relationships and supports, such as direct relatives, neighbours, workmates and friends, are represented in the questionnaire. After the first summary of scores, a distinct familial (parents, spouse, grandparents, children and other relatives) and nonfamilial (neighbour, schoolmate, workmate, other social or sacral company) support score was created. Answers are provided as four-level Likert items (Cronbach’s α = 0.570) [34–36].

Finally, the shortened form of the Athens Insomnia Scale Inventory (AIS-5) was also recorded to detect mild or severe insomnia. The cut-off score of the AIS-5 is ≥4, which is related to potential insomnia (Cronbach’s α = 0.630) [37].

The data were compared to the Hungarostudy (HS) population. Free-access, nationally representative, face-to-face household surveys are conducted in Hungary every 10 years, and the last survey was conducted in 2013 (*n* = 2000) [38, 39]. Hungarostudy is built from the inventories listed above and contains the BDI, STAI, CSSDS, Devins Illness Intrusiveness Rating Scale, PHQ15 and AIS, along with basic questions about age, sex, education, marital status, religion, and physical status. In HS, further questions about smoking, drinking alcoholic beverages and some questions about the income of the participant are asked. In our inventory, a shorter form from HS 2013 was used; in this manner, the two populations became comparable. Identical questions were compared using the propensity score matching method.

Our results were adjusted to a comprehensive frailty score published by Shi et al. to characterize the relationship between traditional frailty syndrome and cognitive decline [40]. The modified frailty index based on our data included the presence of recurrent angina pectoris, atrial fibrillation, congestive heart failure, chronic coronary disease, diabetes mellitus, hypertension, past myocardial infarction, peripheral vascular disease, stroke or TIA, anxiety (defined by the STAI score), asthma or COPD, depression (defined by the GDS score), cognitive impairment (defined by the MMSE score), malnutrition (BMI < 21) and medication (using ≥5 medications daily). The MMSE categories used in the comprehensive frailty score were applied accordingly with the following cut-off values: 27 points and above, 24–26 points, 21–23 points and less than 21 points.

### Outcomes

The primary outcome of the study was the overall mortality rate. As secondary outcomes, one-year and two-year mortality rates were examined.

### Statistical analysis

Descriptive statistics (means, standard deviations, medians and interquartile ranges) were calculated for all continuous variables. Means and SDs were used for variables with a normal distribution, and the Kolmogorov-Smirnov test and the Shapiro-Wilk test were used to ascertain the type of distribution. For categorical variables, Pearson χ^2^-test was used; nonparametric tests were used for continuous variables, with the Mann-Whitney U test as the default. Categorical variables were calculated from continuous scales, with well-proven cut-off values. Univariate and multivariable logistic regression (Cox regression) models were also used. A Kaplan-Meier analysis with the log-rank and Breslow tests were used to investigate differences in short- and mid-term survival rates. *P* < 0.05 was considered statistically significant. For the statistical analysis, IBM SPSS Statistics 24.0 (SPSS Inc., Chicago, Illinois) with the R plugin (version 3.2.1) for PS matching was used. Forest plots were generated using GraphPad Prism version 8.0.1 software for Windows, GraphPad Software, San Diego, California, USA, www.graphpad.com.

A propensity-matching analysis was performed to compare the vascular population and the Hungarian patient cohort. During propensity score matching, pairs were generated from the HS representative group and the vascular surgical group according to age, sex and place of residence. The balance of baseline covariates between the treated and control groups was evaluated using absolute standardized differences. A value less than 0.1 was considered an acceptable standardized bias. As the pairs were created, identical questions were compared to analyse the differences in psychological attitudes and social states between the general and surgical populations.

## Results

### Descriptive and outcome data

Data from 164 patients were analysed. The mean age of the population was 67.05 years (SD ± 9.49), and 35.97% of the patients were female. In the postoperative period, 20.73% of the patients were admitted to the ICU, and the median length of stay was 1.5 days (IQR 1.0–2.0). The median length of the surgical ward stay was 6.0 days (IQR 5.0–9.0 days). During the follow-up period (1312 days, IQR: 924–1582 days), 42 patients (25.61%) died, the 30-day mortality rate was 0.61% (1 patient), and the 1-year mortality rate was 4.88% (8 patients). The vascular POSSUM score was higher in the nonsurviving group [16 points (IQR: 14.00–18.00) vs. 17 points (IQR: 15.00–22.00), *p* = 0.025]. The nonsurviving group had more previous vascular surgeries (43.44% vs. 66.67%, *p* = 0.009). Lower preoperative haemoglobin levels and higher CRP levels were observed in patients who died during follow-up. Among patients who died, the occurrence of previous stroke (16.39% vs. 26.19%, *p* = 0.162) and psychiatric disorders (4.10% vs. 7.14%, *p* = 0.068) tended to be higher than in surviving patients.

We compared the study population with Hungarostudy patients. After propensity score matching (adjusting participants based on age, sex and place of residence), 159 pairs were created. The vascular surgery patient cohort visited health care facilities more frequently over the last year (26.6% vs. 11.8%, *p* < 0.001). The patient cohort had more intensive social support [CSSDS scores were 20 (15.00–23.00) vs. 23 (19.00–27.00), *p* < 0.001 for the population group and the patient cohort, respectively]. Table 2 shows the comparison between the HS population and the surgical group before propensity matching. Table 2 shows the comparison of socioeconomic characteristics between the population of the Hungarostudy survey and our vascular surgery population after propensity score matching.

Table 2. Comparison between the propensity score-matched pairs (Hungarostudy vs. vascular surgery group, *n* = 159 pairs).

### Main results

According to the traditional MMSE categories (normal range of 24 points or higher), 11.59% of the patients had a cognitive impairment. As a novel cut-off value for the MMSE score (normal range 27 points or higher) was used published by She et al. [40], the prevalence of cognitive dysfunction increased to 25.00%. The minimum MMSE score was 18 points, and the maximum score was 30 points.

**Fig. 1:**
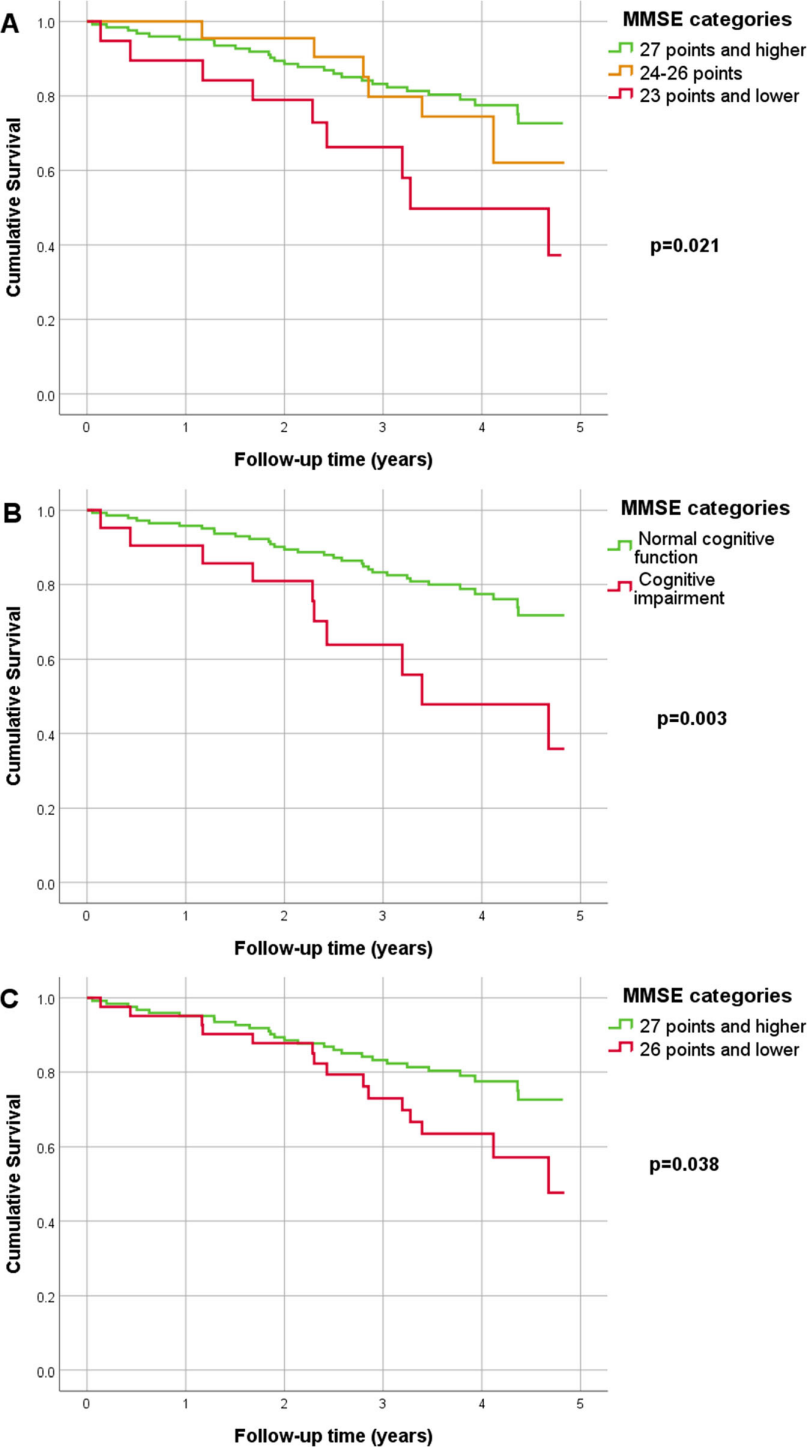
KM curve for MMSE categories and mortality: **a**. MMSE groups: 27 points and higher, 24–26 points, 23 points and lower. **b.** MMSE groups: age and education adjusted normal cognitive function and cognitive impairment. **c**. MMSE groups: 27 points and higher, 26 points and lower (modified cut-off value). Legend: In Fig. 1/A log-rank pairwise comparison was performed: an MMSE score of 27 points or higher vs. 24–26 points, *p* = 0.531; 27 points or higher vs. 23 or fewer points, *p* = 0.007; 24–26 points and 23 points and below, *p* = 0.120

A Kaplan-Meier analysis was performed for survival, and the three curves are shown in Fig. 1. Part A is a summary of the categories used. In Fig. 1/B, age- and education-adjusted cut-off values were applied; in Fig. 1/C, the more sensitive, modified cut-off value was applied as a definition of cognitive dysfunction. All MMSE categories created using the method described above were significantly different fin terms of survival (log-rank *p*-values in Fig. 1, each at the matching Kaplan-Meier curve).

Each worse MMSE –cluster, which was created as described in Fig. 1/A, was associated with an increased risk of long-term mortality adjusted for the vascular POSSUM score (HR: 1.659, 95% CI: 1.129–2.439, *p* = 0.010).

A higher MMSE score exerted a protective effect on all-cause mortality (OR: 0.883, 95% CI: 0.802–0.973, *p* = 0.012). The cohort with cognitive dysfunction (MMSE score ≤ 24 points) had a higher risk of overall mortality after adjustment for the vascular POSSUM score (AHR: 2.918, 95% CI: 1.380–6.170, *p* = 0.005). The one-year survival rate was not significantly affected by cognitive impairment (AHR: 2.360, 95% CI: 0.476–11.692, *p* = 0.293).

**Fig. 2:**
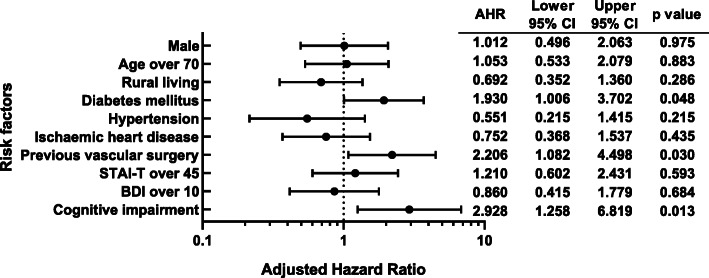
Effects of variables on overall mortality in the multivariate Cox regression model

In addition to these basic factors, the traditional risk factors age- and education-adjusted MMSE scores indicated that cognitive impairment was an important, independent risk factor (AHR: 2.928, 95% CI: 1.258–6.819, *p* = 0.013) in the multivariate Cox regression model. Diabetes mellitus and previous vascular surgery were also independent risk factors for overall mortality (AHR: 1.930, 95% CI: 1.006–3.702, *p* = 0.048 and AHR: 2.206, 95% CI: 1.082–4.498, *p* = 0.030, respectively). The results of the multivariate Cox regression analysis are shown in Fig. 2.

### Other analyses

Self-rated parameters (happiness, satisfaction, and current health status) were lower in the nonsurviving group. The result for happiness was significant (median = 8.0 IQR: 5.0–10.0 vs. 6.0 IQR: 5.0–8.0, *p* = 0.046); furthermore, significance was not reached for satisfaction (median = 7.0 IQR: 5.0–8.0 vs. 6.0 IQR: 5.0–7.0, *p* = 0.122).

BDI, GDS and STAI-T Patient Health Quality 15 and the Caldwell Social Support Dimension Scales were not significantly different between the nonsurviving and surviving groups.

### Relationship between the comprehensive frailty index (FI) and cognitive function

Comprehensive frailty scores were created for each patient. A higher frailty score was associated with a higher risk of mortality (HR: 1.169, 95% CI: 1.022–1.337, *p* = 0.023). The distribution of patients according to the comprehensive frailty index is shown in Table 1.

**Table 1:** Preoperative variables and overall mortality

		All patients (*n* = 164, 100%)						
		Survivors (*n* = 122, 74.39%)			Nonsurvivors (*n* = 42, 25.61%)			
Preoperative Variable		n (%)	Mean/Median	Standard Deviation/ IQR	n (%)	Mean/Median	Standard Deviation/IQR	*p* value^b^
Sex	male	77 (63.11)			28 (66.67)			0.679
Age			66.87	9.98		67.60	7.96	0.874
BMI			27.52	4.72		26.03	3.82	0.092
ASA Score	1	1 (0.82)			0 (0.00)			0.783^c^
	2	46 (37.70)			10 (24.39)			
	3	72 (59.02)			29 (70.73)			
	4	3 (2.46)			2 (4.88)			
Vascular POSSUM^a^			16.00	(14.00–18.00)		17.00	(15.00–22.00)	0.025
*Medical variables*								
Ischaemic Heart Disease		43 (35.25)			15 (35.71)			0.956
Myocardial infarction		23 (18.85)			5 (11.90)			0.302
Diabetes mellitus		35 (28.69)			19 (45.24)			0.049
Obesity		31 (25.41)			5 (11.90)			0.068
Hypertension		108 (88.52)			34 (80.95)			0.214
CABG		10 (8.20)			4 (9.52)			0.791
Previous vascular surgery		53 (43.44)			28 (66.67)			0.009
Stroke or TIA		20 (16.39)			11 (26.19)			0.162
Thyroid disorder		7 (5.74)			2 (4.76)			0.811
Haemoglobin (g/L)			140.33	14.63		129.80	19.70	0.020
Platelet (G/L)			235.37	81.04		251.90	111.90	0.632
Glomerular filtration rate (ml/min)			84.39	13.56		86.20	10.24	0.537
C reactive protein (mg/L)			3.00	(1.16–6.18)		12.35	(4.46–33.50)	< 0.001
*Psychological variables*								
Cognitive dysfunction assessed using the MMSE (age and education adjusted)		11 (9.02)			10 (23.81)			0.013
Depression assessed using the BDI (≥10points)		41 (35.96)			15 (36.59)			0.943
Anxiety assessed using the STAI-T (≥45 points)		47 (38.52)			18 (42.86)			0.621
*Comprehensive Frailty Score* *Comprehensive Frailty Index*			4.00	(3.00–6.00)		5.00	(3.30–6.70)	0.039
1st quartile		47 (38.52)			10 (23.81)			0.201
2nd quartile		20 (16.39)			6 (14.29)			
3rd quartile		35 (28.69)			14 (33.33)			
4th quartile		20 (16.38)			12 (28.57)			

^a^not normally distributed

^b^Pearson chi square test for categorical variables and Mann-Whitney U test for continuous variables

^c^Kolmogorov-Smirnov Z test

## Discussion

### Key findings

In our prospective study, cognitive impairment determined based on the Mini Mental State Examination score was an independent risk factor for postoperative mortality in vascular surgical patients. The MMSE score adjusted for age and education was independently associated with an increased mortality rate. Vascular surgical patients reported more social support and more frequent use of alternative health care facilities than the Hungarian epidemiological population.

Psychological risk factors are components of frailty syndrome, whose relative importance has increased in recent decades [3, 41]. Currently, health care providers have started to recognize that frailty syndromes contain factors in addition to physical domains, such as the daily functioning state, polypharmacy, sarcopenia and other comorbidities [42]. Our article attempts to emphasize the importance of the psychological aspects of frailty syndrome, such as cognitive decline.

A poor functional status and physical frailty have been shown to result in worse postoperative mortality, which are axiomatic statements [43–46]. However, an increasing number of recent studies have focused on the relationship between preoperative cognitive functions and postoperative mortality [40, 47]. According to our recent findings, cognitive dysfunction measured using the MMSE score resulted in worse mid- and long-termsurvival. Nevertheless, a low education level was associated with worse survival in our previous study of patients undergoing cardiac surgery.

The MMSE is an old, well-known and widely used cognitive scale. Although it has advantages, more sensitive tests are currently available to identify mild cognitive impairment (MCI). Nevertheless, the MMSE has great specificity for diagnosing cognitive decline [48]. Studies have reported using modified cut-off values to improve the sensitivity of the test and achieve better applicability to identify cognitive deficits at an earlier stage [17, 18]. For the classification of cognitive functions, we used a different cut-off system for the MMSE score to detect cognitive disabilities in a more precise manner [16]. The traditionally created and modified cut-off values for cognitive impairment groups (MMSE scores of less than 24 in the traditional group and less than 27 in the modified group) were associated with shorter survival.

In contrast, preoperative cognitive deficits did not directly or significantly influence short-term survival. On the other hand, patients with a mild cognitive deficit (MMSE score of 24–26) have a slightly different risk than patients without cognitive dysfunction (MMSE score of 27–30) after approximately 1000 days of follow-up (result shown in Fig. 1). The highest risk of mortality was observed for patients with MMSE scores equal to or less than 23 points.

Previous studies that have noted the importance of depression and anxiety suggest that these mental problems exert an important effect on short- and mid-term survival [49, 50]. However, the severity of depression is identified as an important risk factor, and our current dataset was unable to prove a strong relationship between the observed BDI, GDS or STAI scores with the primary and secondary outcomes. Morin et al. recently published an article in which they concluded that depression severity is a potential predictor of cognitive dysfunction and physical frailty [51]. Our previous study showed a negative correlation between the severity of anxiety and survival in patients who underwent cardiac surgery [50]. According to our recent findings, we hypothesized that cognitive impairment mainly exerted negative effects on mid- and long-term survival in vascular surgical patients.

The comprehensive frailty index (FI) was derived from the study by She et al. and used for our population. Although the original scoring system investigated different frailty domains in cardiac surgical patients [40], our findings revealed similar results. Additional analyses showed that the comprehensive FI also exerts an important effect on overall mortality after vascular surgery.

The other aim of our study was to compare the vascular surgical population to the general, healthy population. The present results are significantly different in several major aspects. After propensity score matching, the analysis clearly shows lower mobility, decreased physical activity and more frequent smoking in the vascular surgical population (Table 2). Several papers have emphasized the importance of social support in different clinical contexts [52–54]. One unanticipated finding was that vascular surgical patients had higher self-reported social support scores. Based on our findings, people undergoing (vascular) surgery or with any other health problems receive higher levels of social support or at least feel that they do.

**Table 2:** Comparison between the propensity score-matched pairs (Hungarostudy vs. vascular surgery group, *n* = 159 pairs)

		Hungarostudy			Vascular surgery group			
		n (%)	Median	IQR	n (%)	Median	IQR	*p* value
No medical contact - last year^a^		42 (26.58)			18 (11.76)			< 0.001
Actual bodily pain^a^		85 (53.46)			85 (53.46)			0.545
Self-reported health condition (1–10)			3.00	(3.00–4.00)		3.00	(3.00–3.00)	0.471
Patient Health Quality			21.00	(16.00–26.00)		20.00	(17.00–24.00)	0.637
Devins Illness Intrusiveness Rating Scale			32.50	(26.00–39.00)		19.00	(13.00–27.00)	0.109
Life satisfaction (1–10)			7.00	(5.00–8.00)		7.00	(5.00–8.00)	0.472
Happiness (1–10)			7.00	(5.00–8.00)		7.00	(5.00–9.00)	0.119
In-hospital-days - last year			0.00	(0.00–0.00)		1.00	(0.00–10.00)	< 0.001
Alternative health care - last 3 years^a^		4 (2.53)			18 (11.32)			0.002
Caldwell Social Support Dimension Scale			20.00	(15.00–23.00)		23.00	(19.00–27.00)	< 0.001
Caldwell Social Support Dimension Scale - family			10.00	(8.00–12.00)		12.00	(10.00–15.00)	< 0.001
Caldwell Social Support Dimension Scale - other			9.00	(7.00–12.00)		10.00	(7.00–13.00)	0.001
Smoking	Never	74 (46.54)			23 (14.74)			< 0.001
	Former smoker	44 (27.67)			80 (51.28)			
	Active smoker	41 (25.79)			53 (33.97)			
Pack years			28.50	(17.50–40.00)		23.00	(13.75–40.00)	0.411
Physical exercise/week			5.00	(4.00–7.00)		2.00	(0.00–6.00)	< 0.001
Other, non-sport physical activity/week			3.00	(1.00–4.00)		1.00	(1.00–4.00)	< 0.001
Drinking alcoholic beverages (1–5)			2.00	(1.00–4.00)		2.00	(1.00–3.00)	0.310
Not religious^a^		50 (32.05)			75 (47.17)			0.024
Education level^a^	Primary school	9 (5.66)			7 (4.40)			0.375
	Secondary school	30 (18.87)			25 (15.72)			
	High school	97 (61.01)			89 (55.97)			
	College	23 (14.47)			38 (23.90)			
Family stage^a^	Unmarried, without partner	7 (4.43)			4 (2.53)			0.002
	Unmarried, with partner	2 (1.27)			7 (4.43)			
	Married	74 (46.54)			88 (55.35)			
	Married but living alone	3 (1.90)			15 (9.49)			
	Divorced, without partner	17 (10.76)			8 (5.06)			
	Divorced, with partner	8 (5.06)			31 (19.62)			
	Widow, without partner	46 (29.11)			5 (3.16)			
	Widow, with partner	1 (0.63)			0 (0.00)			
Number of persons in the same household			2.00	(1.00–2.00)		2.00	(1.00–2.00)	0.618
Financial difficulties^a^		28 (18.18)			19 (11.95)			0.083

^a^categorical variable, the chi square test was used for the statistical analysis; the Mann-Whitney U test was used for continuous variables

### Limitations of the study

A main limitation of the present study is the relatively small sample size. In some aspects, we did not have an adequate number of participants to obtain the appropriate statistical power. The questionnaire took 60 min to complete, which was the cause, while 30% of the potential candidates refused to participate.

This single-centre experience prompted us to be more careful when interpreting our findings.

Another limitation is the lack of mapping of patients’ physiological frailty and physical states. Our patient pool generally has poor mobility. Usually, the low level of mobility is caused by a minimal (sometimes zero) effort threshold to pain due to extensive arterial circulation insufficiency. Thus, exact physical and functional parameters were not recorded.

## Conclusions

The purpose of the current study was to determine the relationship between novel preoperative risk factors based on patients’ psychological and sociological variables and mortality following vascular surgery. After an extended analysis, we identified a significant relationship between the patients’ preoperative cognitive dysfunction and worse long-term mortality. The MMSE was used to assess cognitive impairment with modified cut-off values to obtain a more sensitive estimate. Based on our findings, cognitive mapping should be applied to estimate the postoperative mortality risk more accurately in the future. The presence of the mildest cognitive impairment in the preoperative period potentially represents a risk factor for increased mid- and long-term mortality after vascular surgery.

During the analysis of socioeconomic characteristics, the vascular surgery group reported significantly higher social support than the general control group, as measured using the Caldwell Social Support Dimension Scale. Remarkable associations between psychological and social parameters and the frailty index were shown, which could be a basis of further investigations.

## Data Availability

The datasets used and/or analysed during the current study are available from the corresponding author upon reasonable request.

## Abbreviations

AHR: Adjusted hazard ratio
AIS: Athens Insomnia Scale
ASA: American Society of Anesthesiology
BDI: Beck depression inventory
BMI: Body mass index
CABG: Coronary artery bypass grafting
COPD: Chronic obstructive pulmonary disease
CRP: C reactive protein
CSSDS: Caldwell Social Support Dimension Scale
FI: Frailty index
GDS: Geriatric Depression Scale
HS: Hungarostudy
ICU: Intensive care unit
IQR: Interquartile range
MMSE: Mini Mental State Examination
PHQ: Patient Health Questionnaire 15
SD: Standard deviation
STAI: State Trait Anxiety Inventory
TIA: Transient ischaemic attack
vascular POSSUM: Vascular Physiological and Operative Severity Score for the enUmeration of Mortality and Morbidity
